# Increase in Axial Compressibility in a Spinning Van der Waals Gas

**DOI:** 10.3390/e23020137

**Published:** 2021-01-22

**Authors:** Yun Liu, Hao Liu, Zhen-Guo Fu, Weimin Zhou

**Affiliations:** 1 Department of Applied Physics, School of Physics and Electronics, Hunan University, Changsha 410082, China; yunliu@hnu.edu.cn; 2 Dengjiaxian Innovation Research Center, Institute of Applied Physics and Computational Mathematics, Beijing 100088, China; fu_zhenguo@iapcm.ac.cn; 3 Science and Technology on Plasma Physics Laboratory, Laser Fusion Research Center, China Academy of Engineering Physics, Mianyang 621900, China; zhouwm@caep.cn

**Keywords:** axial compressibility, equation of states, Van der Waals gas, molecular dynamics

## Abstract

We investigated the adiabatic compression along the axial direction of a spinning Van der Waals gas by applying theoretical analysis and molecular dynamics (MD) simulations. Based on the analytical results, the rotation-induced compressibility increase effect is significant in a Van der Waals gas, while the attraction term in the Van der Waals equation of states (EOS) contributes significantly to the compressibility increase in a spinning system. We conducted MD simulations to the axial compression of a spinning gas, whose state is far from the ideal gas state, and further demonstrated that the rotation-induced compressibility increase effect in a dense state is robust, implying that such a phenomenon can be detected in experiments under high-energy-density conditions.

## 1. Introduction

High compression of matter is a fundamental subject in the fields of inertial confinement fusion (ICF) [1,2,3,4], high-energy-density physics [5,6], and laboratory astrophysics [7], including accretion disks or compact stars [8]. The investigation of the compressibility of a spinning gas may particularly be promising significant in the research in related fields, especially in areas such as the design of Z-pinch experiments [9,10], and the applications of devices to improve the efficiency of engines while reducing pollutants [11,12]. 

Geyko and Fisch [13] conducted a theoretical study around the axial adiabatic compression of an ideal spinning gas, demonstrating that part of the external pdV work used to compress the gas converts into the rotational kinetic energy $E_{r}$ of the system during the compression, resulting in lower temperature T and axial pressure p and a softer spinning gas to be compressed.

The ideal gas model is a highly simplified model where interactions between gas molecules are ignored. This raises the question of whether the unusual compressive feature derived from the ideal gas can still be suitable for a nonideal gas. In our previous theoretical work [14], we examined the axial compressibility of a spinning gas using a simple virial equation of states (EOS) and showed that the effect of rotation-induced compressibility increase exists in a spinning nonideal gas with a virial EOS. However, the virial form of EOS can only simply consider the repulsive interactions between gas molecules. In comparison, the Van der Waals EOS includes the modification to the ideal-gas EOS by the repulsive and attractive interactions of gas molecules, so it can better reflect the compressive feature of real gases in a spinning system. Furthermore, as the compression ratio of gas further increases, all the simple theoretical form of EOS will become invalid. The molecular dynamics simulation method can provide an accurate description of the compression process under the conditions far from the ideal gas. Therefore, in order to explore whether the real gas can have the similar compressive properties in a spinning system, we concentrate on the axial adiabatic compression of a spinning Van der Waals gas with the aid of theoretical analysis and numerical molecule dynamics simulation methods.

To keep the conditions consistent with the previous theoretical work, we consider Geyko’s assumptions for the gas compression: (i) the length L of the cylinder is much larger than the radius R of the cylinder so that the end effect (the influence caused by the end surface of the cylinder) can be ignored; (ii) the cylindrical side surface (r = R) is smooth and frictionless; (iii) the compression process is quasi-static; and (iv) the gas is in a thermodynamic equilibrium state throughout the compression process.

A dimensionless equation of the density distribution is derived based on the EOS of a Van der Waals gas, as well as the mechanical equilibrium condition. Here, the density distribution form of the Van der Waals gas is represented by three dimensionless parameters that correspond to the intensity of the rotation, repulsive effect (volume effect) of the gas molecules, and attraction between the molecules. We show the influence of each dimensionless parameter on the compressive features of the gas by comparing the variations of the thermodynamic quantities of the system during compression, under different combinations of the initial dimensionless parameters.

Compared with the theoretical method, the molecular dynamics (MD) simulation method can more accurately describe the behaviors of real gases from the perspective of microscopic particle dynamics [15]. Thus, we simulate the axial adiabatic compression of gases with different initial states using the MD method and show that the spinning gas, far from the ideal gas state, would still exhibit an obvious compressibility increase effect, verifying the robustness of this effect in nonideal gases.

The rest of the article is divided into three sections. In Section 2, we present the theoretical analysis based on the EOS of the Van der Waals gas and discuss the numerical results. The details and results of the MD simulation are provided in Section 3. In Section 4, we outline the main conclusions and a short summary of the findings. The definitions of all parameters and their expressions can be found in Table A1 of the Appendix A.

## 2. Theoretical Analysis

The EOS of a Van der Waals gas is represented by

(1) $$\begin{array}{c} \;\left(p+an^{2}\right)\left(1-bn\right)\;=nk_{B}T, \end{array}$$

where p, n, and T denote the pressure, number density, and temperature of the gas; $k_{B}\;$ is the Boltzmann constant; and parameters a and b correspond to the magnitude of the weak attraction between particles and modification of the molecular volume (thus, b has the dimension of a volume), respectively. In this study, we assume that the values of a and b are constant and do not change with the compression.

Considering the distribution of n in a spinning gas:

(2) $$\begin{array}{c} \;dp=\left\{k_{B}T\left[\frac{1}{1-bn}+\frac{bn}{{\left(1-bn\right)}^{2}}\right]-2an\right\}dn, \end{array}$$

this uses the isothermal approximation. The balance between the centrifugal force dF and dp leads to:

(3) $$\begin{array}{c} \;\left\{\left.k_{B}T\left[\frac{1}{1-bn}+\frac{bn}{{\left(1-bn\right)}^{2}}\right]\;-\;2an\right\}\right.\frac{dn}{n}=mw^{2}rdr, \end{array}$$

where *m* is the mass of one gas molecule, ω is the angular speed, and r is the radius from the axis of the cylinder. Integration of Equation (3) yields:

(4) $$\begin{array}{c} \;k_{B}T\left[ln\frac{n}{n_{0}}-ln\frac{1-bn}{1-bn_{0}}+\frac{1}{1-bn}-\frac{1}{1-bn_{0}}\right]-2a\left(n-n_{0}\right)\;=\frac{1}{2}mw^{2}r^{2}, \end{array}$$

where $n_{0}\;$ is the number density at r=0. We introduce the dimensionless parameters α, ξ, and φ to Equation (4) to obtain a dimensionless expression:

(5) $$\begin{array}{c} \;ln\frac{\tilde{n}}{{\tilde{n}}_{0}}-ln\frac{1-\xi \tilde{n}}{1-\xi {\tilde{n}}_{0}}+\frac{1}{1-\xi \tilde{n}}-\frac{1}{1-\xi {\tilde{n}}_{0}}-\alpha \left(\tilde{n}-{\tilde{n}}_{0}\right)=\varphi {\tilde{r}}^{2}, \end{array}$$

where

(6) $$\begin{array}{c} \;\bar{n}=\frac{N}{V}=\frac{N}{pR^{2}L}, \end{array}\;\begin{array}{c} \;\tilde{n}=\frac{n}{\bar{n}}, \end{array}\;\begin{array}{c} \;\tilde{r}=\frac{r}{R}, \end{array}\;\begin{array}{c} \;\alpha =\frac{2a\bar{n}}{k_{B}T}, \end{array}\;\begin{array}{c} \;\xi =b\bar{n}, \end{array}\;\begin{array}{c} \;\varphi =\frac{mw^{2}R^{2}}{2k_{B}T}. \end{array}$$

Here, we define *N* as the total number of particles in the cylinder with volume V, radius R, and height L. Accordingly, $\bar{n}$ is the mean number density of the system, and {$\tilde{n}$, $\tilde{r}$, α, ξ, φ} is a set of dimensionless parameters denoting, respectively, the number density, cylindrical radius, ratio of the mutual attraction of potential and thermal energies between the gas particles, effect of particle volume (repulsion between the particles), and ratio of the spinning kinetic energy to the thermal energy. We can determine the value of $n_{0}$ in Equation (5) using the equation of the normalized condition:

(7) $$\begin{array}{c} \;\int_{0}^{1}2\tilde{n}\left(\tilde{r}\right)\tilde{r}d\tilde{r}=1, \end{array}$$

this can be applied to Equation (5) to find the distribution of $\tilde{n}\left(\tilde{r}\right)$. Meanwhile, the dimensionless pressure is given by

(8) $$\begin{array}{c} \;\tilde{p}=\frac{p}{\bar{n}k_{B}T}=\frac{\tilde{n}}{1-\xi \tilde{n}}-\frac{\alpha {\tilde{n}}^{2}}{2}, \end{array}$$

whereas the average pressure on the two end surfaces of the cylinder (axial pressure) can be calculated using the equation:

(9) $$\begin{array}{c} \;\bar{p}=\bar{n}k_{B}T\int_{0}^{1}\tilde{p}\left(\tilde{r}\right)2\tilde{r}d\tilde{r}. \end{array}$$

To ensure that the Van der Waals model can work in the calculations of $\tilde{n}(\tilde{r}$), limiting the range of the dimensionless parameters α and ξ is necessary. Firstly, the value of ξ must not exceed 1; the pressure diverges as ξ approaches 1 and the EOS of a Van der Waals gas is invalid when ξ > 1. Secondly, α/ξ < 27/4 should be satisfied during the calculations to ensure the monotonic relationship between the pressure and the number density. When α/ξ is too large, the system becomes dominated by the attraction term between the particles and undergoes a phase transition from a gaseous to condensed state, which is beyond the scope of the model of the Van der Waals EOS.

Now, we consider a continuous, adiabatic, axial compression process of the gas in the cylinder. All parameters of the system, such as $\bar{n}$, T, and ω, will change accordingly when L is decreased. The relationship between $\bar{n}$ and *L* is determined by Equation (6), while *T* and ω vary with L according to the conservation equations of angular momentum and energy. By decomposing the continuous compression process by steps, we can calculate the value of the compression in the spinning gas using these numerical methods.

Suppose that, in a tiny compression, the height of the cylinder changes from L to $L^{\prime }$ and the values of the system parameters $\bar{n}$, T, and ω to ${\bar{n}}^{\prime }$, $T^{\prime }$, and $\omega^{\prime }$, respectively, the angular momentum of the spinning gas is given by:

(10) $$M=mL\int_{0}^{R}2\pi rdrn\left(r\right)\omega r^{2}=2\pi mLR^{4}\omega \bar{n}\int_{0}^{1}\tilde{n}\left(\tilde{r}\right){\tilde{r}}^{3}d\tilde{r}\;\begin{array}{c} \;=2mR^{2}\omega N\int_{0}^{1}\tilde{n}\left(\tilde{r}\right){\tilde{r}}^{3}d\tilde{r}. \end{array}$$

The conservation of angular momentum of the system requires that the angular momentum before and after the tiny compression *M*’ to be equal:

(11) $$2mR^{2}\omega N\int_{0}^{1}\tilde{n}\left(\tilde{r}\right){\tilde{r}}^{3}d\tilde{r}=2mR^{2}\omega^{\prime }N\int_{0}^{1}{\tilde{n}}^{\prime }\left(\tilde{r}\right){\tilde{r}}^{3}d\tilde{r}.$$

The internal energy U could be represented as a function $U\left(T,V\right)$. Choosing T and V as the basic state parameters, we can express the full differential of *U* as:

(12) $$\;dU={\left(\frac{\partial U}{\partial T}\right)}_{V}dT+{\left(\frac{\partial U}{\partial V}\right)}_{T}dV,$$

where the form ${\left(\partial U/\partial T\right)}_{V}=C_{V}$, which represents the isometric heat capacity of the gas and could be substituted into the above equation. Meanwhile, the relationship between p and the Helmholtz free energy *F* is given by

(13) $$-p\;={\left(\frac{\partial F}{\partial V}\right)}_{T}={\left(\frac{\partial \left(U-TS\right)}{\partial V}\right)}_{T}={\left(\frac{\partial U}{\partial V}\right)}_{T}-T{\left(\frac{\partial S}{\partial V}\right)}_{T}.$$

Substituting the Maxwell relation ${\left(\frac{\partial S}{\partial T}\right)}_{T}={\left(\frac{\partial_{p}}{\partial T}\right)}_{V}$ into Equation (13), we have:

(14) $${\left(\frac{\partial U}{\partial V}\right)}_{T}=T{\left(\frac{\partial p}{\partial T}\right)}_{V}-p.$$

Furthermore, substituting Equations (1) and (14) into (12) yields the expression of dU for a Van der Waals gas:

(15) $$dU=C_{V}dT+aNdn.$$

Assuming that $C_{V}=3/2Nk_{B}\;$ for a Van der Waals gas is the same as that of an ideal gas, we can integrate Equation (15) to re-express the internal energy U as:

(16) $$U=\frac{3}{2}Nk_{B}T+aNn$$

where the first and second terms correspond to the thermal energy $E_{t}$ and potential energy $E_{p}$, respectively. Considering the density distribution in the spinning gas, the total potential energy is an integral expression:

(17) $$E_{p}=\int_{0}^{R}an^{2}L2\pi rdr=2aN\bar{n}\int_{0}^{1}{\tilde{n}}^{2}\tilde{r}d\tilde{r}.$$

Energy conserved during the compression is given by pdV=dE. Moreover, we can approximate the whole pdV work during a tiny axial compression of the spinning gas by applying the trapezoidal integral:

(18) $$pdV\approx \frac{\left(\bar{p}+{\bar{p}}^{\prime }\right)\left(V-V^{\prime }\right)}{2},$$

where $\bar{p}$, *V* and ${\bar{p}}^{\prime }$, *V*’ represent the axial pressures and volumes before and after the tiny compression, respectively. Three energies contribute to the total change in the system energy dE, whereas the conservation of energy during the compression is represented by:

(19) $$\frac{\left(\bar{p}+{\bar{p}}^{\prime }\right)\left(V-V^{\prime }\right)}{2}=\Delta E_{t}+\Delta E_{p}+\Delta E_{r},$$

where $\Delta E_{t}$, $\Delta E_{p}$, and $\Delta E_{r}$ correspond to the change in the thermal, potential, and rotational energies of the system during the tiny compression, respectively, which can be calculated individually using the formulas:

(20) $$\Delta E_{t}=\frac{3}{2}Nk_{B}\left(T^{\prime }-T\right),$$

(21) $$\Delta E_{p}=2aN\left({\bar{n}}^{\prime }\int_{0}^{1}{\tilde{n}}^{\prime }{}^{2}\tilde{r}d\tilde{r}-\bar{n}\int_{0}^{1}{\tilde{n}}^{2}\tilde{r}d\tilde{r}\right),$$

(22) $$\Delta E_{r}=\frac{1}{2}M^{\prime }\omega^{\prime }-\frac{1}{2}M\omega .$$

Accordingly, we can find the value of $T^{\prime }$ and $\omega^{\prime }$ after the tiny compression by solving the simultaneous equations of angular momentum (Equation (11)) and energy conservation (Equation (19)) using an iterative algorithm, as well as the distribution of $n\left(r\right)$ and axial pressure. Decomposing the continuous axial compression into a series of tiny compressions, we can generate a variation of the various physical quantities involved in the process through numerical calculations.

**Figure 1:**
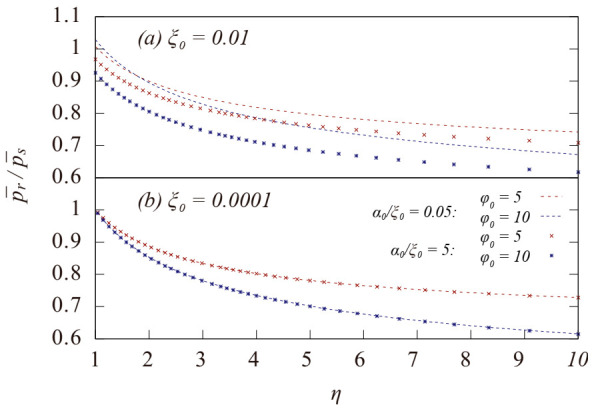
Variation of the pressure ratio of ${\bar{p}}_{r}$ to ${\bar{p}}_{s}$ during an adiabatic compression for a Van der Waals gas system with different initial $\xi_{0}$ and $\alpha_{0}$. $\eta =\bar{n}/{\bar{n}}_{0}$ is the compression ratio. The red and blue dash line correspond to the results with $\alpha_{0}/\xi_{0}=\;0.05$ (weak attraction). The red and blue dots correspond to the result with $\alpha_{0}/\xi_{0}=\;5\;\left(\mathrm{strong}\;\mathrm{attraction}\right)$.

Here, we use the axial pressure ratio ${\bar{p}}_{r}/{\bar{p}}_{s}$, where ${\bar{p}}_{r}$ and ${\bar{p}}_{s}$ are the axial pressure of the spinning system and static system during the compression with the same initial T and $\bar{n}$, respectively, to qualitatively measure the increase in axial compressibility caused by the rotation of the gas. When ${\bar{p}}_{r}/{\bar{p}}_{s}$ < 1, the axial compressibility of the spinning gas is larger than that of the static gas at the same compression ratio. Figure 1 illustrates the variation of ${\bar{p}}_{r}/{\bar{p}}_{s}$ during an adiabatic axial compression with different initial $\alpha_{0}$, $\xi_{0}$, and $\varphi_{0}$.

**Figure 2:**
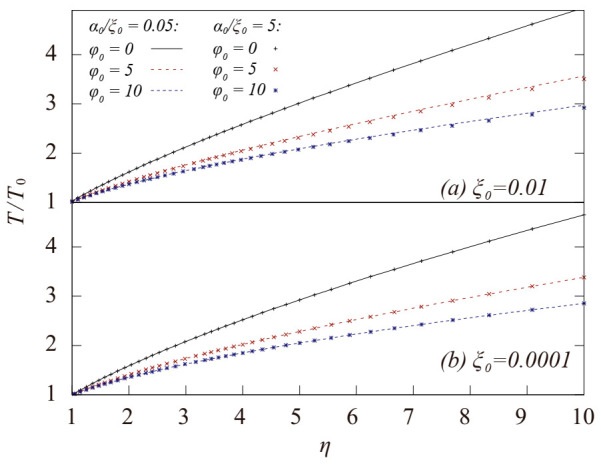
Variation of the temperature ratio of T to $T_{0}$ during an adiabatic compression of a Van der Waals gas system with different initial $\xi_{0}$ and $\alpha_{0}$. $T_{0\;}$ is the initial temperature. The red and blue dash line correspond to the results with $\alpha_{0}/\xi_{0}=\;0.05$ (weak attraction). The red and blue dots correspond to the result with $\alpha_{0}/\xi_{0}=\;5\;\left(\mathrm{strong}\;\mathrm{attraction}\right)$.

For all the curves listed in Figure 1, an axial pressure ratio of less than 1 can be achieved when the compression ratio η ($\eta =\bar{n}/{\bar{n}}_{0}$) is not too large. Moreover, it reaches about 0.6 at a large compression ratio, indicating that the effect of rotation-induced compressibility increase is very significant in our cases. In the case with $\xi_{0\;}$ = 0.01 and $\alpha_{0}/\xi_{0}$ = 0.05, the initial axial pressure ratio is greater than 1, implying that the rotation of the system increases the difficulty in axially compressing the gas in the initial situation. Nevertheless, this situation is reversed as  η  increases during a continuous compression. Figure 1 also suggests that a faster rotation speed (a larger value of $\varphi_{0}$) leads to smaller ${\bar{p}}_{r}/{\bar{p}}_{s}$ at the maximum compression ratio; thus, the rotation-induced compressibility increase becomes more significant as the system spins faster. The spinning gas becomes more compressible than static gas along the axis direction mainly because that part of the external pdV work is converted into $E_{r}$ during the axial compression, which slows down the temperature rise of the spinning gas as compared to that of the static gas, as shown in Figure 2.

Because the parameter α represents the attraction between the gas molecules, which is beneficial to the compression of the gas for both the spinning and static systems, it is evident in Figure 1 that a larger initial value of α_0_ leads to a smaller ${\bar{p}}_{r}/{\bar{p}}_{s}$ at the maximum compression ratio, indicating that the attraction term in Van der Waals EOS can be more conducive to improving the axial compressibility of the gas in the spinning system. As illustrated in Figure 2, the curves of the temperatures with a larger α rise more slowly than those with a smaller α, which is consistent with the behavior of the axial pressure ratio. The attraction term in Van der Waals EOS is $an^{2}$; thus, the average attraction strength in a system with a nonuniform density distribution will be greater than that in a uniform system given the same average density. Additionally, it can explain the greater contribution of the attraction term in a spinning gas to the increase in the axial compressibility, because the rotation of the gas leads to a large degree of nonuniformity of density in radius.

## 3. Molecular Dynamics Simulations

The compressibility of gases is a common concern in high-energy-density physics, especially in the compression characteristics of gases (plasmas) under high-density conditions. As mentioned in the previous section, there are restrictions on the parameter range applicable to the Van der Waals EOS, which make this theoretical model inapplicable to understand the axial compressibility under high-density conditions. On the contrary, these parameter restrictions do not apply to MD methods, where more detailed dynamic processes in the gas compression can be considered. Thus, we employed the MD method to investigate the axial compression of a spinning Van der Waals gas and to further verify the robustness of the effect of rotation-induced compressibility increase in a system close to a real gas. Particularly, we applied the MD method, under the code LAMMPS [16], to simulate the axial compression of a spinning helium gas in a cylinder. The interaction potential between atoms utilized is the well-known Lennard–Jones (LJ) potential (LJ126 form):

(23) $$\;V\left(r\right)=\varepsilon \left[{\left(\frac{\sigma }{r}\right)}^{12}-{\left(\frac{\sigma }{r}\right)}^{6}\right],$$

where $\varepsilon /k_{B}$ = 10.2 K and σ  = 2.28 Å [17].

To avoid the end effect and to satisfy the requirements of assumption (i) given earlier, we set a periodic boundary condition along the axis of rotation. Moreover, the interaction between the atoms and the sidewall of the cylinder was a short-range LJ potential to confine the atoms within the cylinder and to ensure that no friction would be applied on the atoms and the angular momentum of the system could be conserved.

We simulated the compression of the spinning gases in two different initial states to show the difference in the gas behavior near and away from an ideal gas state, which we will refer to as cases I and II. In case I, the initial mean number density was set to ${\bar{n}}_{0}$ = 2.6875 × 10^25^ m^−3^, corresponding to a molar volume of 22.4 L under the standard state; initial temperature to $T_{0}$ = 300 K; and mass of the helium atom at m  = 4 g/mol. Moreover, the cylinder had infinite length due to the boundary conditions. The height of the simulation cell was 1000 Å and contained 84,000 atoms. In case II, the initial mean mass density was set to ρ = 0.125 g/cm^3^ (corresponding to ${\bar{n}}_{0}$ = 7.525 × 10^28^ m^−3^), which is equal to the density of liquid helium, while the initial temperature was still $T_{0}$ = 300 K to ensure that the system was in a dense gas state. However, the cylinder had a radius R = 100 Å and a height $L_{0}$ = 200 Å and contained 120,000 atoms. In both cases, the maximum compression ratio was η = 10.

Here, we introduce the coupling factor $\Gamma =E_{p}/E_{t}\;$ to describe how far the gas deviates from the ideal gas. The value of $\Gamma_{0}$ and $\Gamma_{f}$, corresponding to the coupling factors of the system before and after compression, respectively, are shown in Table 1. Note that during the entire compression process of case I, Γ is very small, implying that the gas could be approximated as an ideal gas. In case II, however, the value of Γ varies greatly during the compression process, approaching 1 after compression, indicating that the potential energy of the interaction between the helium atoms is comparable to the kinetic energy of the thermal motion; thus, the gas is far from the ideal gas state.

The results of theoretical analysis suggest that $\varphi =m\omega^{2}R^{2}/2k_{B}T\;$ is an important dimensionless parameter reflecting the spinning effect. To verify this, we conducted a series of simulations with different initial values of $\varphi_{0}$, with φ = 0 as a reference case.

Note that the density of the rotating system is not uniform under thermodynamic equilibrium. When a rotational angular velocity is applied to a static system in a thermodynamic equilibrium state, the atoms in the system will move outward under the action of a centrifugal force, causing the rise in the system temperature. Assuming that the temperature of the stationary system is $T_{0}$, the temperature rises to $T_{1}$ after the work of the centrifugal force reaches the thermodynamic equilibrium again. From a more comprehensive perspective, if we want to show that the compressibility of the gas is reduced after it has entered the rotating state, we can opt to compare the difference between the static system of $T_{0}\;$ and the rotating system of $T_{1}$ in the compression process as a more reasonable solution than considering two systems with the same initial temperature. To maintain the same conditions as the previous work [8], we temporarily ignored the effect of the gas entering the rotating state process.

**Figure 3:**
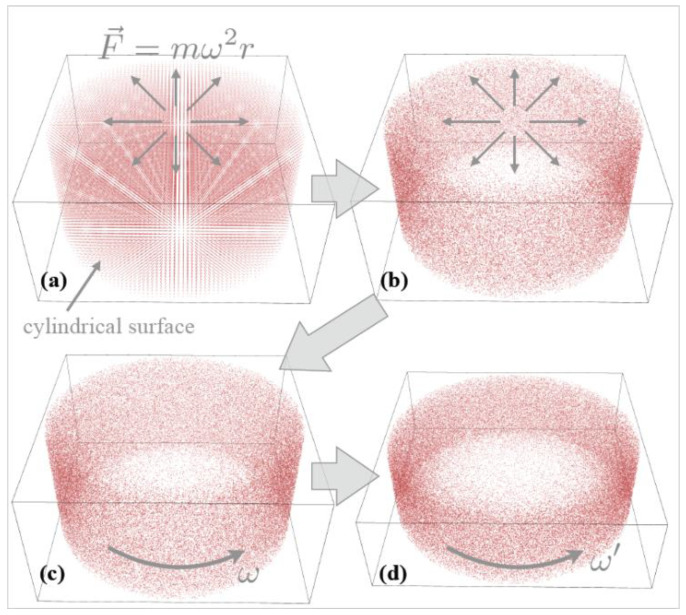
Schematic of MD simulations. (**a**) Initial lattice configuration of the atoms. (**b**) The thermodynamic equilibrium state under centrifugal external field. (**c**) The state-of-rotation equivalent to the centrifugal external field. (**d**) The state after a continuous compression.

As the temperature of the system increased from the static to the rotating state, we needed to ensure that the system was balanced at the set temperature (300 K) after entering the rotating state in simulations. Thus, we followed this procedure in the simulations. First, we applied an equivalent centrifugal external field ${\vec{F}}_{r}=m\omega^{2}\vec{r}$ to all atoms in the stationary system and used a nose-hoover heat bath [18] to allow the system to achieve equilibrium at 300 K under the centrifugal external field. Next, we removed the centrifugal external field and added a velocity component $\vec{v}=\vec{\omega }\times \vec{r}$ to the velocity component of each molecule to achieve the transition from a system under an equivalent centrifugal external field to a real spinning system, as illustrated in Figure 3. Because the radial density distribution of the spinning system is the same as that under an equivalent centrifugal external field, this transition process does not cause a change in temperature.

**Figure 4:**
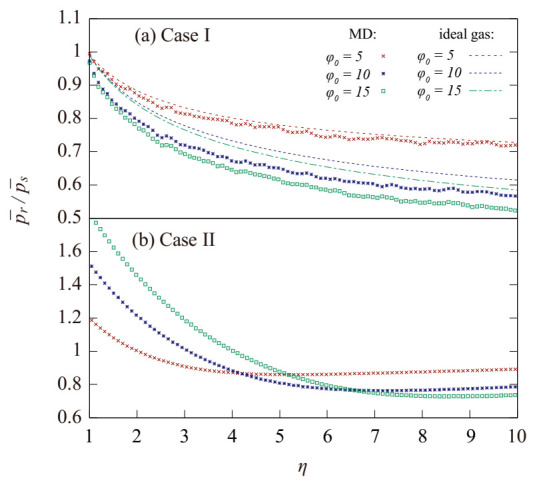
Variation of the pressure ratio ${\bar{p}}_{r}$ to ${\bar{p}}_{s}$ during an adiabatic compression with different initial $\varphi_{0}$ in the MD simulations. $\eta =\bar{n}/{\bar{n}}_{0}$ is the compression ratio. The red, blue, and green dash line correspond to the results in ideal gas. The red, blue, and green dots correspond to the result obtained with MD simulations.

As shown in Figure 4, the value of Γ in case I was very small; thus, the variation of pressure in the spinning system during the continuous compression process was similar to that of the ideal gas. A comparison of the curves would clarify that the value of ${\bar{p}}_{r}/{\bar{p}}_{s}$ in the MD simulations was lower than in the ideal gas, implying that the compressibility increase caused by rotation in the simulations was even larger than that of an ideal gas, especially for the cases with $\varphi_{0}$ = 10 and $\varphi_{0}$ = 15. This phenomenon could be attributed to the attraction term in the LJ potential. 

**Figure 5:**
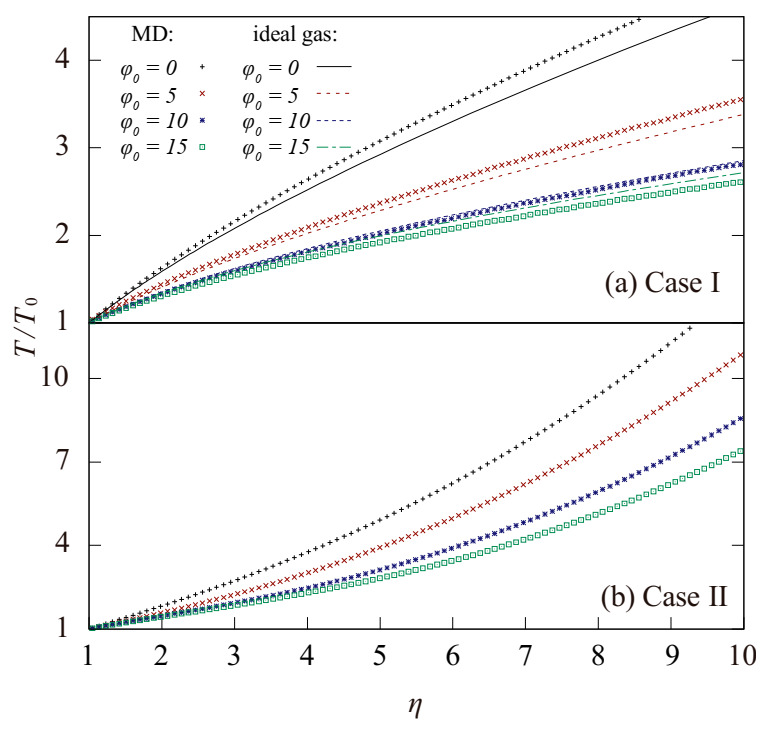
Variation of the temperature ratio T to $T_{0}$ during an adiabatic compression with different initial $\varphi_{0\;}$ in the MD simulations. $T_{0}$ is the initial temperature. The red, blue, and green dash line correspond to the results in ideal gas. The red, blue, and green dots correspond to the result obtained with MD simulations.

Figure 5 shows a comparison of the variation of temperature of the MD simulations during the compression with different initial values of $\;\varphi_{0\;}$ in case I and that of the ideal gas model. For the static system ($\varphi_{0\;}$ = 0), the temperature of MD system rose faster than that of the ideal gas system, suggesting that a larger proportion of the pdV work used to overcome the virial pressure $p_{v}$ during the compression has been converted into the thermal energy $E_{t}$. By contrast, the temperature rises in the MD simulations for the cases with $\varphi_{0\;}$ = 10 and $\varphi_{0\;}$ = 15 was less than that of the ideal gas model. As a result, the value of ${\bar{p}}_{s}$ in the static system, used as a reference value in Figure 4, was larger in the MD simulations than in the stationary ideal gas, whereas ${\bar{p}}_{r}/{\bar{p}}_{s}\;$ in the simulations were lower than in the ideal gas system.

As also depicted in Figure 4, the initial pressure increase caused by the rotation was very obvious in case II. Here, a larger value of $\varphi_{0}$ corresponded with a larger initial pressure ratio ${\bar{p}}_{r}/{\bar{p}}_{s}$. Moreover, as the value of η increased, the ${\bar{p}}_{r}/{\bar{p}}_{s}\;$ of the systems with a larger $\varphi_{0\;}$ decreased at a faster rate and eventually exceeded those for the systems with a smaller $\;\varphi_{0\;}$, which resulted in a smaller ${\bar{p}}_{r}/{\bar{p}}_{s}$ similar to that for the virial gas model [9].

The variation of temperature in case II is shown in Figure 5. Larger values of the initial $\varphi_{0}$ resulted in a slower temperature rise during the compression, similar to other cases mentioned in this work, except that the proportion of the system temperature increase $T/T_{0}$ during compression was much higher. As η reached 10, $T/T_{0}$ was lower than 5 for the stationary ideal gas, whereas it was larger than 10 in case II with $\varphi_{0\;}$ = 0 and $\varphi_{0\;}$ = 5, mainly because a large part of the pdV work overcoming the virial pressure during compression was converted to $E_{t}$ for the gas systems having $E_{p}$ comparable to $E_{t}$, which accelerated the increase in the temperature during compression. Generally speaking, the potential energy of the system showed a very strong nonlinear relationship with the thermodynamic parameters of the system, which entails that the presence of potential energy in nonideal gases will lead to unpredictable behavior of the system during compression.

The energy conversion relationship during the entire compression process is shown in Table 1. Note that, as the value of $\varphi_{0\;}$ increased, the proportion of the pdV work converted to the rotational energy $\gamma_{r}$ increased incrementally, whereas the proportion converted to thermal energy $\gamma_{t}$ decreased incrementally, making the compressibility reduction caused by rotation more pronounced. Moreover, the proportion of the pdV work converted to potential energy $\gamma_{p}$ did not change much with the change of $\varphi_{0\;}$ for both cases I and II.

## 4. Summary

We applied the theoretical analysis and MD methods to investigate the axial compression of a spinning Van der Waals gas in a smooth cylinder. Based on the analytical results and MD calculations, the Van der Waals gas exhibited a rotation-induced compressibility increase effect during a continuous axial compression; the primary reason is that the temperature of the spinning gas rising more slowly than that of the stationary gas. We also found that the attraction term in the Van der Waals EOS had a significant contribution to the axial compressibility increase in the spinning system. Moreover, our MD simulations for a spinning gas with a large coupling factor Γ showed that the increase effect of the rotation-induced compressibility remained significant even when the gas state was far from the ideal gas state, which further illustrates the robustness of this effect. The robustness of this effect indicates that it is likely to be observed in experiments. Furthermore, this effect can be applied in many fields where high compression of gas or plasmas is required, such as Z-pinch experiments. The facility of Z-pinch experiments is a cylinder. The increase in compressibility is helpful to achieve the experimental goal of compressing high-temperature plasma to an extremely high density and areal density.

In the theoretical analysis and simulation of this work, the assumptions adopted are difficult to realize under a real compression of real gas. The effects of non-equilibrium dynamics during a non-quasi-static compression, wall friction and non-adiabatic process on the experimental results should be taken into consideration in further investigations.

## Figures and Tables

**Table 1:** Parameters of the molecular dynamics (MD) simulations during the compression. $\Gamma =E_{p}/E_{t}\;$ is the coupling parameter of the gas, with $\Gamma_{0}$ and $\Gamma_{f}$ corresponding to the coupling parameters of the initial and final state. Similarly, $\gamma_{p}$, $\gamma_{r}$ and $\gamma_{t}$ are the proportion of the changes in the potential energy $\Delta E_{p}$, rotational energy $\;\Delta E_{r}$ and thermal energy $\Delta E_{t}$, respectively, and correspond to the total pdV work during the whole compression.

	φ_0_	$\Gamma_{0}$	$\Gamma_{f}$	$\gamma_{p}$	$\gamma_{r}$	$\gamma_{t}$
				(%)	(%)	(%)
Case I	0	1.58 × 10^−5^	3.29 × 10^−4^	0.04	0	99.96
	5	1.31 ×10^−5^	3.96 × 10^−4^	0.04	18.33	81.63
	10	6.15 × 10^−5^	7.13 × 10^−4^	0.07	31.76	68.13
	15	1.21 × 10^−5^	1.11 × 10^−4^	0.11	35.68	64.20
Case II	0	0.0112	0.750	44.74	0	55.26
	5	0.0283	0.823	45.17	4.82	50.01
	10	0.0219	0.927	42.66	16.34	40.99
	15	0.0996	1.012	39.05	27.12	33.83

## Data Availability

The datasets used or analysed during the current study are available from the corresponding author on reasonable request.

## Appendix A

**Table A1:** The list of definitions and expressions of all parameters.

Parameter	Definition	Expression
p	pressure	
n	the number density	
T	temperature	
$k_{B}$	Boltzmann constant	
a	coefficient of the weak attraction term in Van der Waals EOS	
b	coefficient of the molecular volume modification in Van der Waals EOS	
m	mass of a gas molecule	
ω	the angular speed	
r	the dimensionless radius	
N	the total number of particles	
$\bar{n}$	the mean number density	$\frac{N}{pR^{2}L}$
$\tilde{n}$	the dimensionless number density	$\frac{n}{\bar{n}}$
$\tilde{r}$	the cylindrical radius	$\frac{r}{R}$
α	the dimensionless parameter describing the ratio of the attractive interaction energy to the thermal energy of the system	$\frac{2a\tilde{n}}{k_{B}T}$
ξ	the dimensionless parameter describing the strength of molecular repulsion potential	$b\bar{n}$
φ	the dimensionless parameter describing the ratio of the spinning kinetic energy to the thermal energy	$\frac{m\omega^{2}R^{2}}{2k_{B}T}$
V	the volume of the cylinder	
R	the radius of the cylinder	
L	the height of the cylinder	
M	the angular momentum	
U	the internal energy	
$C_{V}$	the isometric heat capacity	${\left(\frac{\partial U}{\partial T}\right)}_{V}$
$\Delta E_{t}$	the change in the thermal energy	
$\Delta E_{p}$	the change in the potential energy	
$\Delta E_{r}$	the change in the rotational energy	
${\bar{p}}_{r}$	the axial pressures of the spinning system	
${\bar{p}}_{s}$	the axial pressures of static system	
${\bar{n}}_{0}$	the initial mean number density	
η	the compression ratio	$\frac{\bar{n}}{{\bar{n}}_{0}}$
